# Oropharyngeal Probiotic ENT-K12 as an Effective Dietary Intervention for Children With Recurrent Respiratory Tract Infections During Cold Season

**DOI:** 10.3389/fnut.2022.900448

**Published:** 2022-05-10

**Authors:** Hongyan Guo, Xiaochen Xiang, Xuan Lin, Qiang Wang, Si Qin, Xinyan Lu, Jiawei Xu, Ying Fang, Yang Liu, Jing Cui, Zhi Li

**Affiliations:** ^1^Medical College, Wuhan University of Science and Technology, Wuhan, China; ^2^Institute of Infection, Immunology and Tumor Microenvironment, Hubei Province Key Laboratory of Occupational Hazard Identification and Control, Medical College, Wuhan University of Science and Technology, Wuhan, China; ^3^Department of Endocrinology, CR & WISCO General Hospital Affiliated to Wuhan University of Science and Technology, Wuhan, China; ^4^College of Food Science and Technology, Hunan Agricultural University, Changsha, China

**Keywords:** *Streptococcus salivarius* subsp. *thermophilus* ENT-K12, recurrent respiratory tract infections, oropharyngeal probiotics, children, dietary intervention

## Abstract

Recurrent respiratory tract infections (RRTi) cause a high burden of disease and lead to negative impact on quality of life, frequent school/work absenteeism, and doctor visits, which remain a great challenge to pediatricians because RRTi can increase the risk of various complications including antibiotic overuse and resistance, which is one of the biggest threats to global health, and there is no confirmed effective treatment. In this study, we aimed to assess the clinical efficacy and safety of oropharyngeal probiotic ENT-K12 as a dietary intervention or a complementary treatment along with standard medical treatment during acute respiratory infections among children with RRTi during cold season. The results of this study show that when comparing to practicing of standard medical treatment only, the complementary intake of oropharyngeal probiotic ENT-K12 can effectively reduce episodes of both acute and RRTi in school children, shorten the course of respiratory symptoms onset, reduce the use of antibiotics and antiviral drugs, and reduce the absence days from both children's school and parents' work. Using oropharyngeal probiotics as a complementary dietary intervention to stabilize oropharyngeal microflora, specifically inhibiting respiratory pathogens and enhancing host immunity, could possibly be a promising approach to reduce RRTi burden and combating antibiotic resistance in long term, more clinical studies will be needed to further confirm the clinical practicing guide to ensure its clinical benefit.

**Methods:**

A total of 100 susceptible children with RRTi aged 3–10 years, living in Wuhan, China, were selected. They were randomized to the probiotic group and control group at the beginning of the trial during the cold season. Fifty children in the probiotic group took oropharyngeal probiotic ENT-K12 for 30 days, along with standard medical treatment when there was an onset of respiratory symptoms and medical treatment was needed, and fifty children in the control group did not take oropharyngeal probiotics but only had standard medical treatment when there was an onset of respiratory symptoms and medical treatment was needed. Patients were followed up for 30 days during the cold season. The primary objective of this study is to assess the complementary dietary interventional efficacy of oropharyngeal probiotic ENT-K12 on episodes of respiratory tract infections during the cold season, and the secondary objective is to assess the interventional efficacy of oropharyngeal probiotic on days of respiratory symptoms onset, using antiviral drugs, antibiotics, and antipyretics, days of children absent from school, and days of parents absent from work, as well as to confirm tolerability and safety judged by adverse event reporting.

**Results:**

There were 47 children, 22 male and 25 female children, with an average age of 5.71 years (SD = 1.99) in the probiotic group finishing the study, and 50 children, 32 male and 18 female children, in the control group with an average age of 6.12 years (SD = 1.98) finishing the study. During the 30-day period of oropharyngeal probiotic intake, children in the probiotic group totally had 7 episodes of upper respiratory tract infections, while children in the control group totally had 17 episodes of upper respiratory tract infections, indicating that the incidence of upper respiratory tract infection in the probiotic group (14.89%) was significantly lower than that in the control group (34.00%) during the intervention period. The days of using antibiotics and antiviral drugs in the probiotic group were significantly lower than that in the control group, and the course of respiratory symptoms onset was shorter and more moderate in the probiotic group than that in the control group; in addition, compared with the control group, both the days of children absent from school and parents' absence from work in the probiotic group were significantly lower. Children treated with oropharyngeal probiotic ENT-K12 had excellent tolerability with no side effects reported, hence confirmed safety of applying oropharyngeal probiotic ENT-K12 as a prophylactic use or an effective dietary intervention along with standard medication during respiratory infections onset.

**Conclusion:**

Intake of oropharyngeal probiotic ENT-K12 as a dietary intervention can effectively reduce episodes of upper respiratory tract infections in school children with RRTi during high peak season, reduce the days of using antibiotics and antiviral drugs, and reduce children's sick leave days, parents' absence days from work, and shorten the course of respiratory infections; the safety of oropharyngeal probiotic ENT-K12 has been confirmed with no side effects reported, excellent tolerability, and easy acceptance. Notably, this study opens up a new research idea in the field of microbe promoting human health by supplying direct proof to support its efficiency and safety.

## Introduction

The oropharyngeal microflora acts as a barrier against colonization of potentially respiratory pathogens and against overgrowth of already present opportunistic respiratory pathogens. A balanced and stabilized oropharyngeal microflora can help control the growth of opportunistic respiratory pathogens and coordinate with host immune system impacting human health and diseases; in contrast, host colonization with respiratory pathogens and the formation of pathogenic biofilms during childhood cause recurrent respiratory tract infections (RRTi) and chronic inflammations such as wheezing and asthma that are difficult to eradicate in later life (1), and the increased otopathogen abundance and decreased oropharyngeal/nasopharyngeal bacterial diversity that significant increased hazard ratios for allergic diseases are associated with commonly prescribed multiple classes of antibiotics and viral RRTi during infancy (2, 3), a cross-sectional survey conducted in Beijing, China, with 7,222 preschool-aged children revealed that asthma, allergy, initial use of antibiotics before 6 months, and breast feeding might exert a graded, dose-dependent effect on RRTi susceptibility, especially the significant odds of RRTi were 8.31 for asthma, which plays a leading role (4). Compared with healthy adults, infants and young children are at an increased risk of RRTi due to the relative immaturity of their immune system. According to a cohort study, children with RRTi documented a median of 113 days with respiratory symptoms, 9.6 acute respiratory infection episodes, and 6 physician visits for respiratory infection per child per year; early nasopharyngeal colonization with *Streptococcus pneumoniae* was common in children who later developed RRTi, while asymptomatic rhinovirus infections at young age in children with recurrent infections may due to aberrant innate immune responses. Exposure to antibiotic treatment early in life may have a lasting impact on the composition of the microbiota leading to permanent replacement by resistant pathogens. The clinical sequelae of RRTi can result in long-term complications and prolonged antibiotic regimens prescribed and further contribute to the disease economic burden, which is projected to increase over the next 20 years, highlighting the importance of safe and effective prevention strategies for reducing RRTi prevalence in children (5, 6).

Pooled prevalence of Group A *Streptococcus* from pharyngeal specimens was 37% in children who present with sore throat and 12% in asymptomatic children (7), in China, *S. pneumoniae* colonization was found in 21.6% in children aged under 5 years with fever and flu-like symptoms, of which the frequency is positively correlated with the number of respiratory pathogens detected providing a strong evidence against empiric antibiotic use for treating respiratory infections (8). Comparing respiratory virome and serum cytokine profiles between children diagnosed with acute respiratory tract infections also indicates that respiratory microbe homeostasis and specific cytokines are associated with the onset of RRTi over time (9). In our experience, pediatric outpatient visits caused by RRTi are generally ≤ 10 years of age, and most are treated more frequently with antibiotics and corticosteroids to eliminate acute-onset respiratory symptoms because there is no international consensus about the best effective and safe prophylactic treatment for pediatric RRTi, even if systematic review of the efficacy and safety of immunotherapy for RRTi were reported (10, 11).

Recently, the beneficial aspects of bacteriocins, a set of miscellaneous peptide-based bacterium killers produced by microbiota commensals, have been considered as a backup plan for traditional antibiotic treatments to cure multiresistant infections and finely reshape the endogenous microbiota for prophylaxis use of RRTi (12). Bacteriocins, salivaricin A2, and salivaricin B specifically produced by oropharyngeal probiotic ENT-K12 have been reported to contribute bactericidal activities to various respiratory pathogens, including most *Streptococcus pyogenes* and *Micrococcus luteus* strains (13); the bactericidal mode of action of salivaricin B is to interfere with the cell wall peptidoglycan biosynthesis of respiratory pathogens resulting in the reduction of its cell wall thickness together with changes to cytoplasmic membrane integrity, which kill 40% of *S. pyogenes* cells after 30 min and 90% in <3 h of exposure; the irreversible defection of the cell envelopes leads to a failure to generate daughter cells indicating a rapid killing activity; yet no salivaricin B-resistant *S. pyogenes* strains have been reported previously, and so far no significant resistance to salivaricin B was reported (14, 15). The recent computing study has demonstrated that salivaricin B showed predicted binding affinity toward the ACE2, the binding domain of severe acute respiratory syndrome coronavirus 2 (SARS-CoV-2), with potential activity against the SARS-CoV-2 beta variant (16). The mode of action of bacteriocins is remarkably different from conventional antibiotics, and the machinery used by pathogens to develop resistance should be different. With this in mind, bacteriocins may be considered as new-age infection fighters (17).

A study using real-time PCR to monitor the persistence of oropharyngeal probiotic ENT-K12 administration in oral cavity indicates that within few days of administration, ENT-K12 typically colonizes various sites of oropharyngeal mucosal membranes including the tongue, buccal, and both sides of pharynx; its simulated bacteriocin can be detected in saliva (18), plus that the megaplasmid encoding salivaricin A2 and salivaricin B appear to be transmissible between *S. salivarius* strains with the megaplasmid having been observed to move to another *S. salivarius* strain in the oral cavity of subjects colonized with strain ENT-K12 (19), providing its beneficial effect of maintaining oropharyngeal homeostasis and as an antipharyngitis agent (20, 21), and the ability of preventing immune activation induced by oral pathogens without altering the native salivary microbiome and inflammatory markers in healthy oral cavity (22). Furthermore, oropharyngeal probiotic ENT-K12 is able to downregulate immune responses through the action on NF-κB signaling pathways elicited by pathogens, inhibit NF-κB subunit translocating into the nucleus, attenuate IL-8 secretion induced by inflammatory mediators, upregulate genes responsible for activating the interferon signaling pathways with antiviral and cytokine modulation properties, and specifically alter the expression of host genes involved in multiple innate defense pathways, epithelial layer adhesion, and homeostasis pathways, altogether stimulating an anti-inflammatory response and actively protecting the host from inflammation and apoptosis induced by pathogens (23). This study aimed to confirm the recognition that administration of oropharyngeal probiotic ENT-K12 among children with RRTi could provide complementary therapeutic effects for acute respiratory infections and inflammation, and to prevent new respiratory episodes in children with RRTi, meanwhile to reduce the needs of antibiotic courses for pediatric RRTi.

## Materials and Methods

### General Information

The single-center, open, randomized controlled trial was conducted in Wuhan CR & WISCO General Hospital from January to March 2021 according to the criteria set by the Declaration of Helsinki and with the approval of the local ethics committee, the Ethics Committee of CR & WISCO General Hospital Affiliated to Wuhan University of Science and Technology (registration number HRWGZYY20210003). The parents and their children who participated in the trial were informed of the trial methods and signed the consent.

To be considered legible for enrollment into the study, subjects met the following criteria: (1) children aged 3–10 years who attend school; (2) children who experienced 3 or more episodes of upper respiratory infections in the previous year; and (3) parents/legal guardians who were familiar with and agree to the study and signed informed consent. Subjects were excluded from the study if they met the following exclusion criteria: (1) children with immunological insufficiency or defects; (2) children who underwent tonsillectomy or adenotonsillectomy; (3) children who were suffering from bronchospasm, asthma, allergic rhinitis, adenoid hypertrophy, or chronic sinusitis; (4) children who were allergic to milk protein; (5) children who were suffering from systemic disease, such as diabetes, kidney disease, gastrointestinal diseases, or rheumatology; (6) children who were suffering from infectious diseases such as measles, mumps, or rubella; (7) children who had been taking antibiotics and immunosuppressive drugs in the past week; and (8) children who had on-site symptoms of respiratory tract infections such as sore throat, other upper respiratory infections, or suspected/confirmed respiratory infections.

### Study Material

The study product, Bactoblis oropharyngeal probiotic formula, is formulated in the form of slowly dissolving oral lozenges by Probionet GmbH (Herisau, Switzerland); the preparation of this formula used in the clinical trial contained no < 1 billion colony-forming units (CFUs)/lozenges of *Streptococcus salivarius* ENT-K12 (also known as *Streptococcus salivarius* subsp. *thermophilus* ENT-K12) over shelf life.

All children enrolled in this study underwent a general clinical examination and were randomized to receive oropharyngeal probiotic for 30 days in the probiotic group or no intervention in the control group. Parents/guardians were asked to instruct their children to take one lozenge before bed after brushing their teeth every evening and suck the lozenge until it is fully dissolved (~4–5 min) to ensure that the child does not chew or swallow the lozenges directly. The children were suggested not to drink or swallow any substance for at least 1 h after the administration of the study product.

During the entire study period, if any symptoms of respiratory tract infections are present, the parents of children are required to contact the research practitioner and visit the hospital for further medical examination and medical treatment prescribed if needed; if there was evidence of respiratory tract infections, the enrolled children in both groups were asked to record the variety and duration of drug treatment and continue complementarily taking the study product throughout the study period in case of antibiotic or other medical treatment was required. The participated subjects were required to return for final visits after 30 days and return any unused study product. Compliance will be assessed by counting unused lozenges at the final visit; compliance criteria judged at 90% of dispensed lozenges consumed.

### Objectives

During the study, at least five visits over a 2-month period were involved, namely, screening visit on day 0 (Visit 1), routine visit on days 7 and 14 (Visit 2 and 3), final visit on day 30 (Visit 4), and follow-up visit on day 60 (Visit 5). Additional visits took place if the enrolled children experienced symptoms of respiratory tract infections, so a diagnosis could be confirmed and if necessary, a prescription provided. The primary objective was to access efficacy of oropharyngeal probiotic ENT-K12 on reducing incidence of respiratory tract infections among children with RRTi during cold season. The secondary objective was to assess the efficacy of oropharyngeal probiotic ENR-K12 on the reduced number of days of respiratory symptoms onset, the reduced number of days under treatment with antibiotics, antiviral drugs, antipyretics, and steroids, the reduced number of days the children were absent from school and their parents were absent from work, and to confirm safety judged by adverse events reported. The episodes of upper respiratory tract infections are confirmed after clinically diagnosed according to a child's medical history and symptoms on site.

### Statistical Method

SPSS 26.0 statistical software was used to process the data, and the data were expressed as mean ± standard deviation (X ± S). The chi-square test and Wilcoxon rank sum test were used to compare the general data (i.e., age and gender), and the Wilcoxon rank sum test was used to compare the different clinical outcomes between two groups, and *P* < 0.05 was considered statistically significant. Kaplan–Meier statistics were used to evaluate the level of protection of oropharyngeal probiotics against respiratory tract infections over time. The survival analysis was used to determine the differences in cumulative incidence of patients under different conditions during clinical observation.

## Results

A total of 100 children were selected to participate in this trial. A total of 50 children received oropharyngeal probiotic ENT-K12 every day before bed for 30 days as the probiotic group, and the other 50 children did not receive oropharyngeal probiotics served as the control group. Two children in the probiotic group dropped out of the study for personal reasons at the beginning of the trial; one child in the probiotic group was removed from the trial due to irregular use of oropharyngeal probiotic, and the data were not included in the statistical study. Finally, 47 children in the probiotics group completed the experiment, and 50 children in the control group completed the experiment.


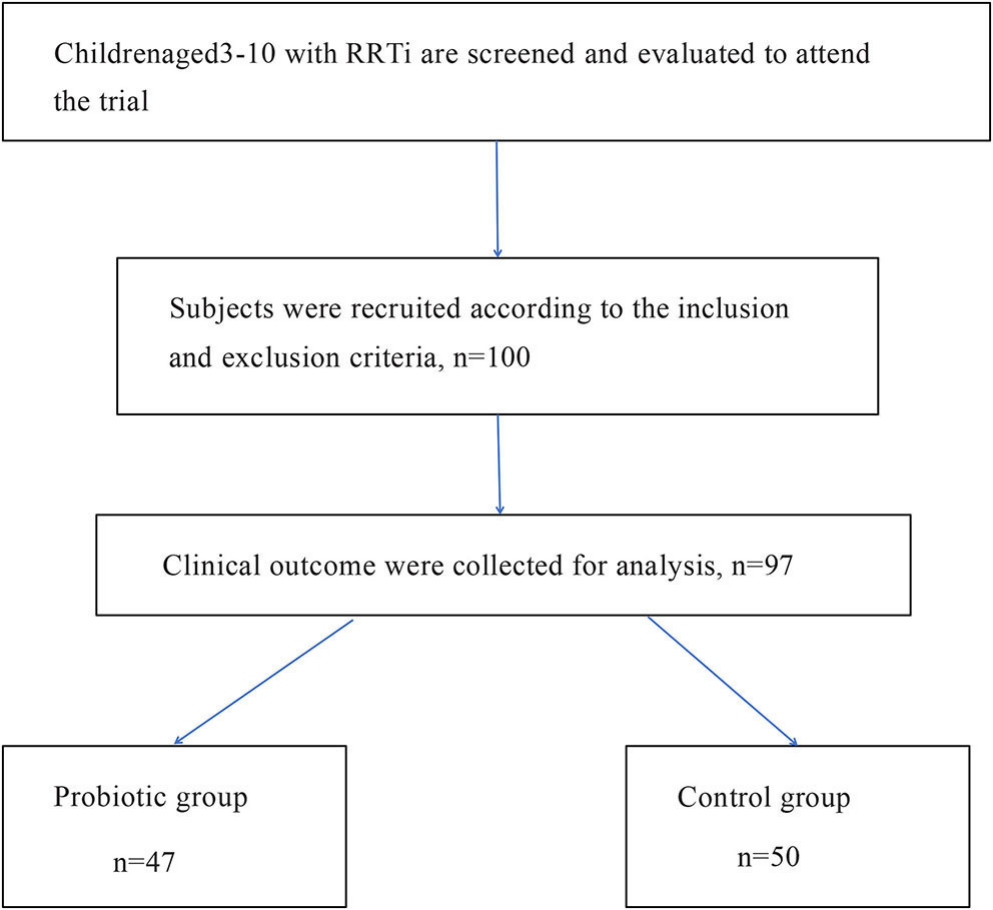


As shown in Table 1, there was no difference in basic characteristics between the two groups. The probiotic group consisted of 22 male and 25 female children, while the control group consisted of 32 male and 18 female children. There was no difference in gender, mean age, the episode numbers of respiratory infections in the past year, pneumococcal vaccination, and influenza vaccination between the two groups (*P* > 0.05).

**Table 1:** The baseline characteristics of children with recurrent respiratory tract infections (RRTi).

	**Probiotic group** **(*n =* 47)**	**Control group** **(*n =* 50)**	***P*** **-value**
Gender			0.228
Male	22	32	
Female	25	18	
Age	5.71 ± 1.99	6.12 ± 1.98	0.301
Numbers of respiratory tract infections in the past year	3.37 ± 0.53	3.58 ± 0.92	0.536
Pneumococcal vaccination			1
No	47	50	
Yes	0	0	
Influenza vaccination			1
No	47	50	
Yes	0	0	

*X ± S is used to describe the average level and variability of the data*.

Table 2-1 shows the clinical outcomes of oropharyngeal probiotic administration among children with RRTi during the 30 days intervention in cold season; oropharyngeal probiotic ENT-K12 administration significantly reduced the episodes of respiratory tract infections by 56% (*P* < 0.05) comparing with the control group, of which, 7 episodes of respiratory tract infections were observed in the group of 47 children with RRTi treated with oropharyngeal probiotic and 17 episodes were observed in the group of 50 non-treated children with RRTi. By comparison with the control group, children with RRTi in the probiotic group experienced a significantly fewer days of respiratory symptoms onset (by 68%, *P* < 0.05), of which a total of 20 days (0.42 days/person) of respiratory symptoms onset observed in the probiotic group, whereas a total of 65 days (1.3 days/person) was observed in the control group. Meanwhile, complementary treatment with oropharyngeal probiotics during the acute respiratory infections along with the standard medication further resulted in a significantly shorter (by 27%, *P* < 0.05) average duration of each respiratory episodes (2.85 days/episode) compared with the control group (3.93 days/episode). Due to the reduction in incidence and duration of each episode, children with RRTi treated with oropharyngeal probiotic had significantly fewer days absent from school by 80% (*P* < 0.05), whereas children treated with oropharyngeal probiotics had averagely 0.26 days/person absence from school and 1.32 days/person was reported in the control group. Apart from children's school attendance, parents' work attendance affected by their children's sickness was also recorded, and the parents of children treated with oropharyngeal probiotic had significantly less days absent from work by 97% (*P* < 0.05); of which 0.02 days/person of absence from work was reported for the probiotic group and 0.6 days/person were reported for the control group. During the study period, when there was evidence of a respiratory tract infection, the enrolled children in both groups were asked to record the variety and duration of medication including antibiotic treatment prescribed by the study practitioner, and continue taking oropharyngeal probiotic ENT-K12 throughout the study period. Our data reveal that children in the probiotic group had significantly less medication compared with those in control group. The number of days taking antibiotics complementarily treated with oropharyngeal probiotics was observed to be reduced by 97% (*P* = 0.05); it is reported that totally 1 day (0.02 days/person) of medication history on antibiotics was observed in the probiotic group compared with totally 32 days (0.64 days/person) observed in the control group; moreover, number of days taking antiviral drugs complementarily treated with oropharyngeal probiotics was observed to be reduced by 90% (*P* < 0.05); it is reported that totally 3 days (0.06 days/person) of medication history on antiviral drugs was observed in the probiotic group compared with totally 30 days (0.6 days/person) observed in the control group; furthermore, no record of antipyretics/steroid intake was observed in the probiotic group compared with 9 days (0.18 days/person) that observed in the control group (*P* = 0.086).

**Table 2-1:** The clinical outcomes during the 30 days of oropharyngeal probiotic intervention among children with RRTi in cold season.

	**Probiotic group** **(*n =* 47)**	**Control group** **(*n =* 50)**	***P*** **-value**
Incidence of respiratory tract infections	7/47 (14.89%)	17/50 (34.00%)	0.045
Respiratory symptoms onset (days)	20	65	
Sick days (days/person)	0.42 ± 1.05	1.30 ± 2.22	0.030
Duration of each episode (days/episode)	2.85 ± 0.69	3.93 ± 2.11	0.034
Absence from school (days/person)	0.26 ± 0.65	1.32 ± 2.33	0.040
Absence from work (days/person)	0.02 ± 0.14	0.60 ± 1.55	0.016
Taking antibiotics (days/person)	0.02 ± 0.14	0.64 ± 1.93	0.052
Taking anti-viral drug (days/person)	0.06 ± 0.32	0.60 ± 1.55	0.045
Taking antipyretics drug (days/person)	0	0.18 ± 0.82	0.086

*X ± S is used to describe the average level and variability of the data*.

Table 2-2 shows the clinical outcomes of oropharyngeal probiotic administration during the 30 days follow-up period in cold season. During the follow-up period, there were no observed episodes of respiratory tract infections among 47 children with RRTi treated with oropharyngeal probiotic, and 6 episodes (incidence 12%, *P* < 0.05) of upper respiratory tract infections observed in 50 children with RRTi of the control group. Similarly, there were no observed respiratory symptoms onset, absence from school or work, and no need for taking standard medication in 47 children with RRTi treated with oropharyngeal probiotic, while 23 days (0.46 days/person, *P* < 0.05) of respiratory symptoms onset, resulting in average 4.2 days per respiratory episodes (*P* < 0.05), 0.46 days per child absence from school (*P* < 0.05), 0.3 days per parent absence from work (*P* < 0.05), 0.16 days per child taking antibiotics (*P* < 0.5), 0.14 days per child taking antiviral drug (*P* < 0.5), and 0.06 days per child taking antipyretics drug (*P* < 0.5) among children with RRTi were observed in control group during the 30 days follow-up period.

**Table 2-2:** The clinical outcomes during the 30 days of follow-up period.

	**Probiotic group** **(*n =* 47)**	**Control group** **(*n =* 50)**	***P*** **-value**
Incidence of respiratory tract infections	0	6/50 (12.00%)	0.025
Respiratory symptoms onset (days)	0	23	
Sick days (days/person)	0	0.46 ± 1.43	0.027
Duration of each episode (days/episode)	0	4.20 ± 1.64	0.027
Absence from school (days/person)	0	0.46 ± 1.29	0.014
Absence from work (days/person)	0	0.30 ± 0.90	0.025
Taking antibiotics (days/person)	0	0.16 ± 0.81	0.164
Taking anti-viral drug (days/person)	0	0.14 ± 0.70	0.164
Taking antipyretics drug (days/person)	0	0.06 ± 0.42	0.327

X ± S is used to describe the average level and variability of the data.

**Figure 1:**
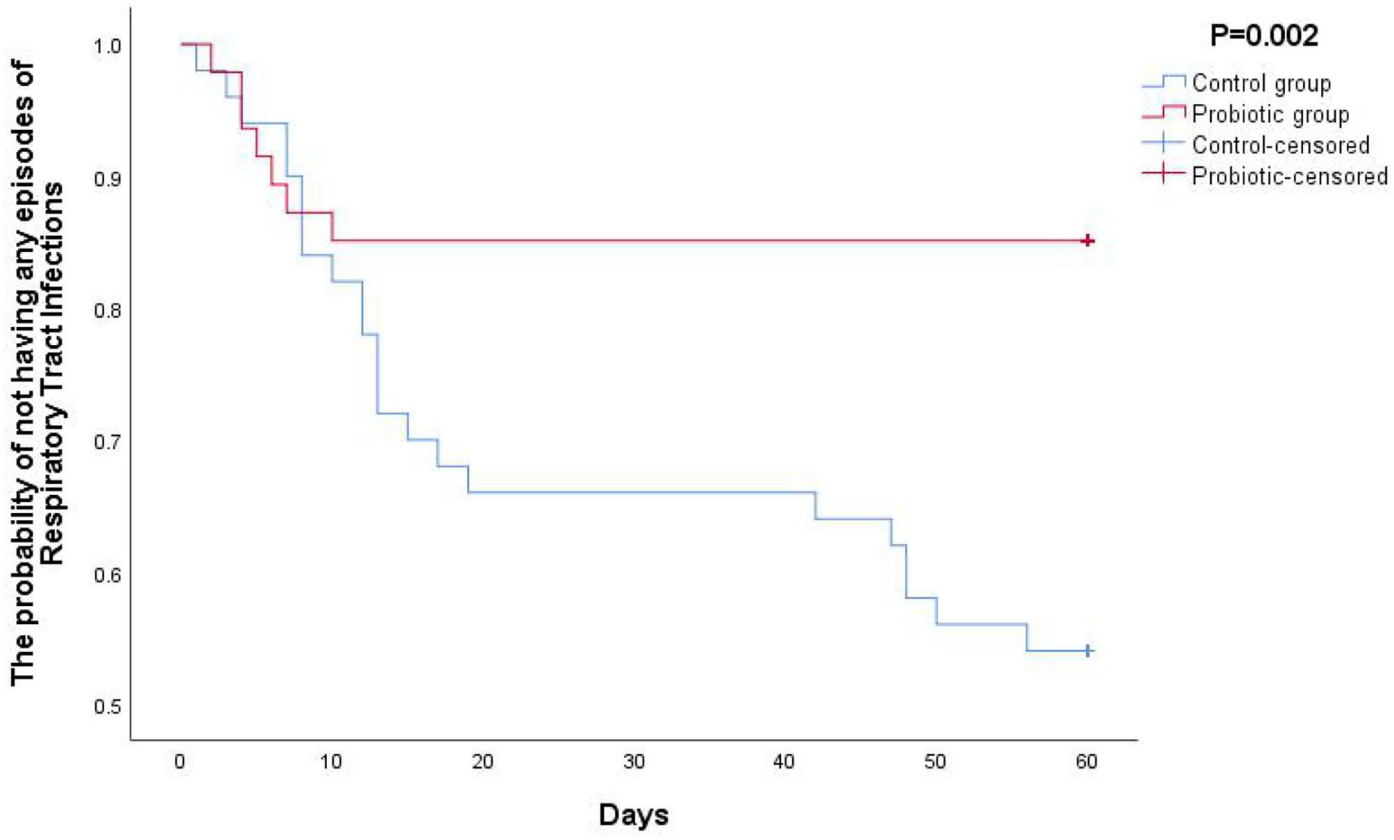
The Kaplan–Meier curve of probability of not having any episodes of respiratory tract infections.

**Figure 2:**
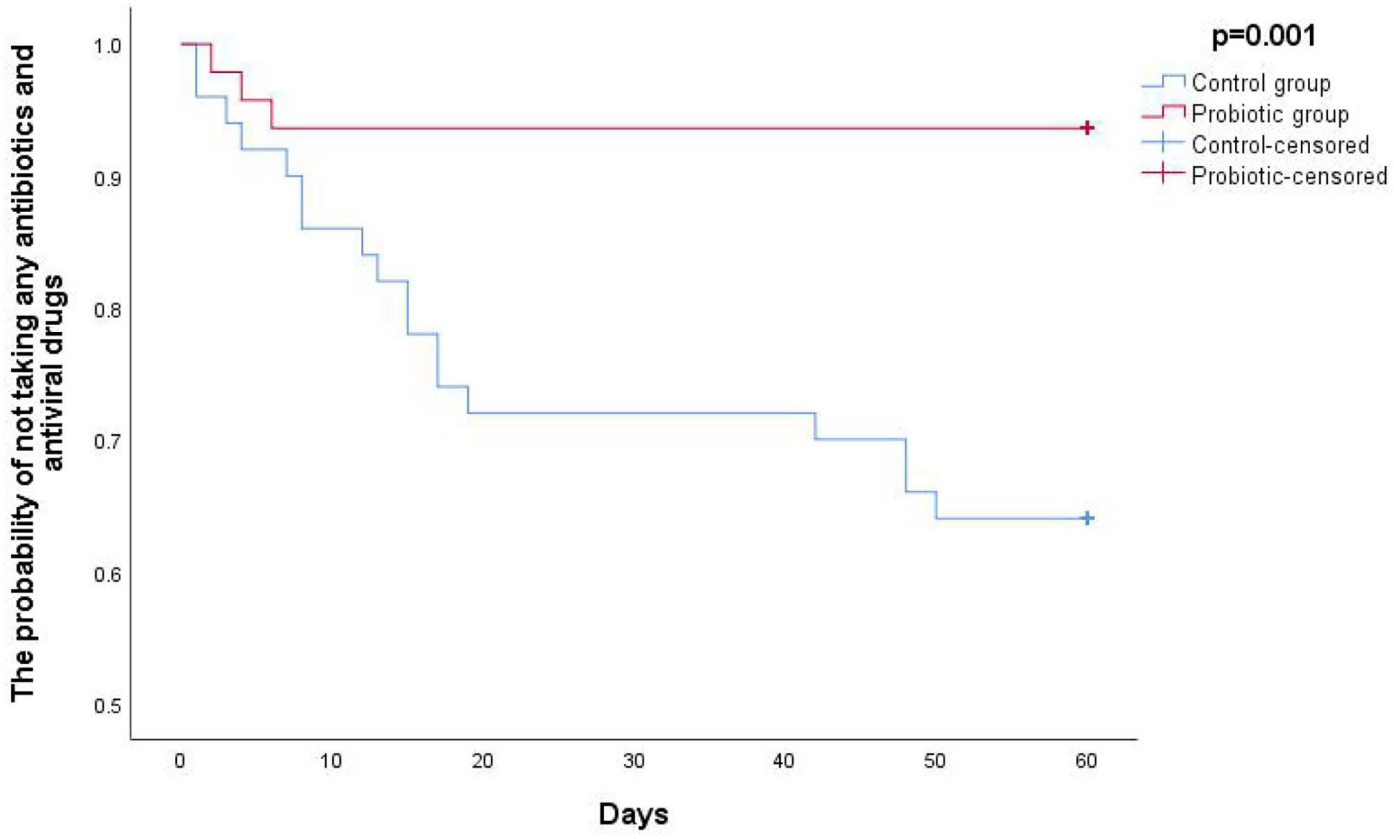
The Kaplan–Meier curve of probability of not taking any antibiotics and antiviral drugs.

Furthermore, the Kaplan–Meier statistical analysis was used to estimate the level of protection effect of oropharyngeal probiotic over time; as shown in Figure 1, the Kaplan–Meier curve of probability not having any episodes of respiratory tract infections decreased gradually from 1 on the first day approaching to a flat at 0.83 on day 12, while the flat lasted until day 60 when this study ended, as per the control group, the probability not having any episodes of respiratory infections continuously decreased to 0.54 at the end of this study (*P* = 0.002). As shown in Figure 2, the Kaplan–Meier statistic is used to estimate the needs of medical treatment including antibiotics and antiviral drugs over time; the probability of not having any courses of antibiotic and antiviral drugs treatment decreased gradually from 1 on the first day approaching to a flat at 0.94 on day 6, while the flat lasted until day 60, the end of this study; in contrast, the probability of not having any courses of antibiotic and antiviral drugs treatment continuously decreased approaching to 0.64 until the end of this study in the control group (*P* = 0.001). Notably, children in the probiotic group experienced sustained protection from respiratory tract infections; hence, the needs for medical treatment were reduced within 2 weeks of oropharyngeal probiotic administration resulting in an extremely lower incidence rate of acute or RRTi and treatment courses comparing with the control group.

Table 3 shows the tolerance and side effects reported by children treated with oropharyngeal probiotic ENT-K12. There is a very high profile in terms of tolerability as 46 out of 47 children had an “excellent feeling” about taking oropharyngeal probiotic ENT-K12 lozenges and another 1 had a “good feeling” about taking oropharyngeal probiotic ENT-K12 lozenges, while there were no side effects reported during the whole study period.

**Table 3:** Tolerance and side effects reported by children treated with oropharyngeal probiotics (*n* = 47).

**Results**	**Tolerability**	**Side-effects**
Excellent, *n*	46	None
Good, *n*	1	None
General, *n*	0	None
Non-qualified, *n*	0	None

## Discussion

Largely assessed data have shown that certain gut probiotic strains may carry a potential to modify the incidence of respiratory infections or duration of each episode (24–27), and in favor of the probiotic groups regarding to incidence of respiratory infections, severity of symptoms, incidence of antibiotic use, and absent days of school having been reported; however, the beneficial levels of those gut probiotics in preventing new respiratory episodes in children with RRTi were limited due to low-quality evidence, and many of the said clinical trials showed no statistically significant difference of illness episodes between intervention and control groups (28, 29). Due to colonization at the interfaces between the environment and both respiratory tract and digestive tract, alone with performing various mode of actions, administration of oropharyngeal probiotics can provide a much greater level of beneficial effects to protect host from respiratory infections comparing to gut probiotics; it was suggested that the oropharyngeal probiotic strain ENT-K12 should be incorporated in future trials of bacteriotherapy of RRTi as it showed outstanding positive influences on human ear-nose-throat health during short-term administration and great safety profile (30–32). Key symptoms of the respiratory tract infections observed during this study include sneezing, fatigue, sore/itchy throat, cough, low fever, nasal congestion, running nose, and dizziness, and there were 3 children who experienced high fever >38°C in the control group during the intervention period, while no children experienced high fever in the probiotic group (data not shown). Considering that most children extremely resist and are scared by the hospital blood collection process, while unnecessary serological diagnosis and etiological examination are not required for commonly happened respiratory infections and common cold, serological diagnosis and etiological examination were not conducted for all the children during this study. In this study, incidence of respiratory infections among children with RRTi reduced 56% during the cold season during the 30 days intervention; if counting on the follow-up period together, the reduction rate would be 68% during the whole study period of 60 days, this is a consistent result with earlier research that oropharyngeal probiotic ENT-K12 treatment in children with a history of recurrent pharyngotonsillitis significantly reduced not only their new episodes by >90% in 3 months compared to the previous year (33) but also during the following 9 months of follow-up period (34), reduced the incidence of both pharyngotonsillitis and acute otitis media in children (35), and significantly reduced the incidence of both upper and lower respiratory tract infections such as tonsillopharyngitis, tracheitis, rhinitis, laryngitis, and otitis media among young children (36). It is worth to mention that during the middle of this study, there was a coronavirus disease 2019 (COVID-19) endemic in Wuhan and the short-term influence was reflected in the incidence of respiratory infections during that time, which can be observed in both Kaplan–Meier curves, an obvious flat exists before day 20 lasting until around day 40; according to the epidemic prevention policy in Wuhan, many communities including the families of the children participated in this study should spontaneously or passively be quarantined for 14 days while people paid much more attention wearing masks at all times in the public in order to stop the spread of COVID-19 endemic ensuring the public health. This evidence prove that not only keeping social distance and wearing masks could effectively protect people from respiratory infections that transmitted *via* droplets and aerosols (37), but also maintaining a balanced oropharyngeal microflora *via* administration of oropharyngeal probiotics could further keep children from various kinds of respiratory infections including SARS-CoV-2 (38), and the said oropharyngeal homeostatic could effectively prevent respiratory infections on occupational health including first-line medical personnel who are in close contact with patients hospitalized for COVID-19 during pandemic (39).

This study may offer some fresh insights into the relationship between the duration of oropharyngeal homeostatic and the incidence of pediatric respiratory infections that reflected the children's immunity status; in this study, the accumulated incident rate of respiratory infections dramatically slowed down since day 12 among children with RRTi treated with oropharyngeal probiotics while it kept increased among children with RRTi in the control group; this phenomenon could possibly explain the colonization of oropharyngeal probiotics in oral cavity, 12 days are estimated for an efficient colonization at the dose of taking 1 lozenge per day, hence providing a better oropharyngeal homeostatic that could probably last for at least 30 days after stop consuming oropharyngeal probiotics; similar phenomenon was also been observed in the previous study among frontline medical staff who fight against COVID-19, without been observed in the control group, medical staff treated with 2 lozenge/day of oropharyngeal probiotic ENT-K12 were effectively been benefited by the oropharyngeal homeostatic since day 10 lasting until the end of study, indicating an effective colonization at dose of 2 lozenges per day (40). Another study also shown that the abundance of otopathogens, *Moraxella*, was lower in the nasopharynx and abundance of oral commensal bacteria, *S. salivarius*, was higher in saliva in young children after taking oropharyngeal probiotic ENT-K12 for 1 month (41), indicating the alteration of oropharyngeal/ nasopharyngeal microflora after oropharyngeal probiotic intervention.

In this study, a total of 7 episodes were observed in the probiotic group during the first 12 days, and only 3 courses of medical treatment were prescribed by the research practitioner during the first 6 days, which indicates that since 1 week after administration, children with RRTi only had minor respiratory symptoms onset while their immune system was able to recover themselves without antibiotic or antiviral drug intervention. This phenomenon was not observed in the control group, while 18 courses of medication were prescribed along with 23 episodes throughout the entire study period; this further explains the fact that a balanced oropharyngeal/nasopharyngeal homeostatic could provide host a better tolerance to respiratory infections, and the upper respiratory microbiome landscape could be considered to be used for medical diagnostics and as a target for therapy (42).

In this study, repeated episodes were not observed in children treated with oropharyngeal probiotic ENT-K12, while 3 repeated episodes (all the repeated episodes were observed during the follow-up period for all 3 children) were observed in the control group, as the number of study subjects and RRTi episodes were small, statistic data are not shown, and it would be interesting to see the difference in new repeated episodes incidence among children with RRTi when conducting a longer period study with a larger sample size.

Previous studies have shown that treatment with oropharyngeal probiotic reduced the exacerbation frequency of chronic adenoiditis and the requirement for medication (43). A similar beneficial effect of oropharyngeal complementary treatment was observed in our study, during intervention period; when acute respiratory symptoms onset, children complementary treated with oropharyngeal ENT-K12 in combination with medication (3 courses in total; 1 took antibiotics for 1 day and 2 took antiviral drugs for 1 and 2 days, respectively) or without medication further shortened the duration of respiratory symptoms onset compared to those treated with only medication in control group (14 courses in total; prescribed with antibiotics, antiviral drugs or antipyretics for various period of time from 1 to 10 days). This complementary beneficial effect was also been demonstrated in previous study that oropharyngeal probiotic treatment among pediatric patients with recurrent pharyngotonsillitis of whom the pharmacological approach was no longer effective thus referred to tonsillectomy surgery could effectively prevent new episodes and reduced the needs for antibiotic treatment and tonsillectomy by 72% comparing to control group (44). It further explains that oropharyngeal administration may improve homeostasis, which plays an important role in host susceptibility to respiratory infections.

Up to date, the international consensus of the available approaches for the prevention of RRTi in children is immunotherapy, while probiotic treatment has not been suggested yet because it is not clear which bacteria strain, dosage, and administration schedule can offer the best results (45). The perspective of “new-age” immunotherapy, such as checkpoint inhibition triggering the immunopathology events or cytokine therapies modulating inflammation development, has been raised to reduce the burden of infectious diseases threatened by antibiotic resistance (46). The recent meta-analysis has shown that the immunostimulants, OM-85 BV and pidotimod, which have been widely used and suggested by pediatricians in the prevention and treatment of RRTi in susceptible children, can reduce the incidence of respiratory tract infections by 0.21 and 0.19 per month, respectively (47); however, long-term use of the said drugs is challenging for children and their families. Actually, probiotics do have great potential as the “new-age” immunotherapy, as several *in vitro*/*in vivo* studies have summarized the strain-specific immunomodulatory effects and the stimulation of interferon (IFN) pathways to antagonize viral respiratory infections due to that microbiome has co-evolved with the eukaryotic genome of its host (26, 48). While waiting for new knowledge, more clinical trials should be conducted to help us having more insights regarding to the clinical benefit of oropharyngeal probiotics administration, alone or complementary use with medication, which could be an effective and safe immunotherapy for children with RRTi in the near future.

The limitations of this study include the lack of oropharyngeal microflora analysis data before and after the intervention of oropharyngeal probiotics as we have limited experiences in oropharyngeal microbiome study methodology. Furthermore, the size of this study was not big enough, and duration was not long enough for us to conduct a statistic analysis on the reduction of RRTi incidence in children treated with oropharyngeal probiotics; however, an obvious trend of RRTi incidence reduction could already been observed in only 30 days and lasted for another 30 follow-up days in this study. Finally, there might be influences on the data of children and parents absent from school and work cause Chinese New Year national holiday was overlapping the later period of this study, and the leave days could have been increased in the control group if it was normal working days; however, a trend already could be observed that 0.07 days of work leave taken was corresponded to each school leave day of children in probiotic group, while 0.45 days of work leave taken was corresponded to each school leave day of children in control group during the intervention period (statistical data not shown), which might further indicate that the days of parents absent from work might associated with the severity of pediatric respiratory symptoms onset.

## Conclusion

There are growing interests in the microbiome-based therapeutics, which provide exciting opportunities for decreasing susceptibility to infections and enhancing resistance to a range of diseases (49); economic studies showed that compared to non-probiotic consumption, generalized probiotic intake in the United States and Canada population would have allowed cost savings for the healthcare, averting respiratory illness sick days and antibiotic prescriptions (50, 51). To develop an effective approach that can be used alone or in combination with antibiotics reducing the dose required for activity is of the upmost importance. Oropharyngeal probiotic ENT-K12 lozenge as a delivery carrier for both innate immune modulator fighting viral infections and bacteriocins showing multiple modes of action by inhibiting cell wall biosynthesis of respiratory pathogens stimulated by ENT-K12 strain, treatment of oropharyngeal probiotic ENT-K12 can effectively prevent new episodes of acute and RRTi in school children with RRTi during cold season, shorten the duration and decrease the severity of respiratory symptoms onset when complementary treated with medication, reduce the use of antibiotics and antiviral drugs, and the absence days from both children's school and parents' work. Using oropharyngeal probiotics as a complementary treatment to stabilize oropharyngeal microflora, specifically inhibiting respiratory pathogens, could possibly be a promising approach to reduce RRTi burden and combating antibiotic resistance in long term, more clinical researches are needed to further confirm the optimized clinical practices.

## Data Availability Statement

The raw data supporting the conclusions of this article will be made available by the authors, without undue reservation.

## Ethics Statement

The studies involving human participants were reviewed and approved by CR & WISCO General Hospital Affiliated to Wuhan University of Science and Technology. Written informed consent to participate in this study was provided by the participants' legal guardian/next of kin.

## Author Contributions

XLI, QW, and SQ guided and completed the whole experimental design. XLU and JX involved in the data collection. HG and XX were responsible for the arrangement of data and analyzing the data. YF, YL, JC, and ZL participated in the interpretation of the results. HG and XX wrote the initial draft with all authors providing critical feedback and edits to subsequent revisions. HG and XX have contributed equally to this work and share first authorship. XLI and QW reviewed and revised the manuscript before submission, they are co-corresponding authors.

## Funding

This research is a self-funded project.

## Conflict of Interest

The authors declare that the research was conducted in the absence of any commercial or financial relationships that could be construed as a potential conflict of interest.

## Publisher's Note

All claims expressed in this article are solely those of the authors and do not necessarily represent those of their affiliated organizations, or those of the publisher, the editors and the reviewers. Any product that may be evaluated in this article, or claim that may be made by its manufacturer, is not guaranteed or endorsed by the publisher.
